# Supplementary material for: Nonlinear molecular dynamics of quercetin in Gynocardia odorata and Diospyros malabarica fruits: Its mechanistic role in hepatoprotection
Source: PLoS One. 2022 Mar 21;17(3):e0263917. doi: 10.1371/journal.pone.0263917 (PMC8936497; doi:10.1371/journal.pone.0263917)
Supplement: S1 File — (PDF) [file pone.0263917.s001.pdf]

Electronic supplementary file 1

Nonlinear Molecular Dynamics of Quercetin in *Gynocardia odorata* and *Diospyros malabarica* fruits: Its mechanistic role in hepatoprotection

Arabinda Ghosh[1†], Pranjal Sarmah[2†], Harun Patel[3], Nobendu Mukerjee[4], Rajbardhan Mishra[5], Saad Alkahtani[6**], Rajender S.Varma[7], Debabrat Baishya[2*]

DPPH and H_2O_2 scavenging assay

The antioxidant activities of the fruit extracts along with standards were assessed based on the radical scavenging effect of stable DPPH. A solution of DPPH of concentration 0.2 mM was prepared in 70% methanol and kept overnight. Stock solution (1mg/mL) of the extract was prepared in 70% methanol. Varying concentrations 10, 20, 50, 100, 150, 200, 300, 400 and 500 µL were prepared in different test tubes and the volume was made up to 1000 µL. 1 mL DPPH was added to each solution and kept at dark for 30 minutes. Ascorbic acid and gallic acid were taken as standards. Optical density of these samples was measured at 517 nm along with blank where 1 mL methanol with 1 mL DPPH solution was taken. All the assays were recorded in triplicates and the values were expressed as Mean± S.D.

Histopathological study of liver

Livers were removed and preserved in 10 (%, v/v) formalin solution. Histopathological analysis was done on the H/E-stained mounted slide preparation of the livers. The histopathological study was carried out in accordance with OECD guidelines (OECD guidance document on Histopathology for inhalation studies, 28th September 2009 Draft).

Results:

Antioxidant activity of the fruit extracts

The antioxidant activity of fruit extracts was measured in terms of its IC_{50} values. Against DDPH and H_2O_2, the free radical scavenging activity of *D. malabarica* and *G. odorata* were displayed in figure 1 (A-D). The IC_{50} values of *D. malabarica* and *G. odorata* was found to be lowest against DPPH 8.68 and 18.97 µg/mL, respectively (Figure 1A and 1B), and with H_2O_2 6.45 and 11.88 µg/mL, respectively (Figure 1C and 1D), which indicates that they have a high antioxidant activity.

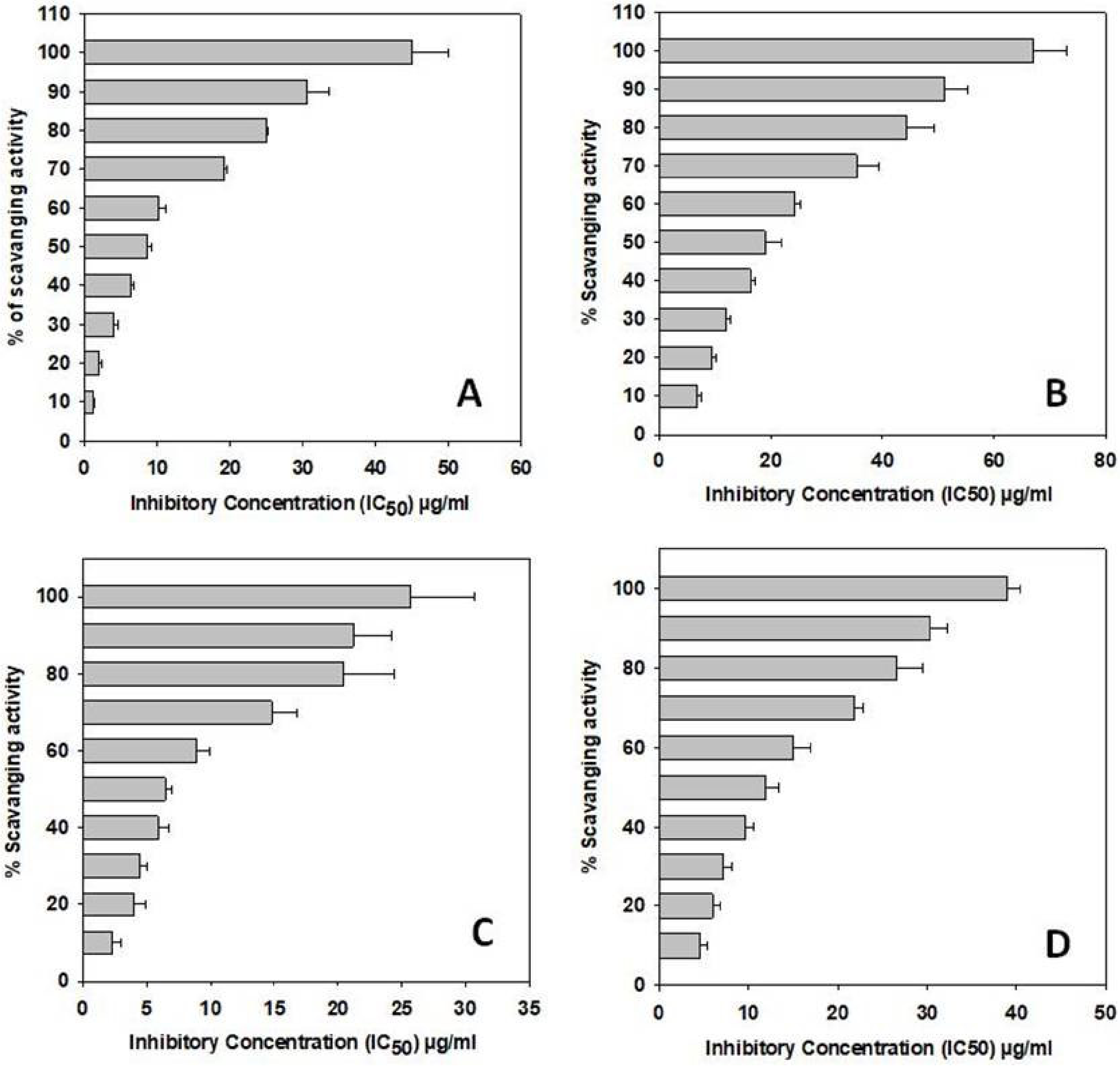

Figure S1. Free radical antioxidant activities of methanolic fruit extracts of against DPPH (A) *D. malabarica*, (B) *G. odorata*, and against H_2O_2 (C) *D. malabarica*, and (D) *G. odorata.*

Histopathological study

The transverse sections of liver tissues of all the experimental groups of Wistar mice have been displayed in figure S2 (A-E). In the control transverse section of the liver (Goup A) showed healthy tissues (Figure S2A), in contrast the hepatocytes displayed the appearance of polynuclei with granular cytoplasm in the negative control (Group B) treated with CCl_4 that signify the induction of liver injury (Figure S2B). In Group C, regeneration of hepatocytes around the blood vessels was observed in the silymarin treated liver (Figure S2C) but granular appearance still persists. Appearance of newly formed hepatocytes signified the hepatoprotective action of commercial drug silymarin. Interestingly, Group D and Group E rats displayed newly formed hepatocytes around the blood vessels and comparatively low granular structures than silymarin treated liver of Group C rats. Groupd D and Group E rats were treated with the purified quercetin molecule from *D. malabarica* and *G. odorata*, respectively (Figure S2D and S2E). Therefore, form this study it could be envisaged that the active quercetin ingredient in the fruit extracts of *D. malabarica* and *G. odorata* induce hepatoprotection against CCl_4 liver injury.

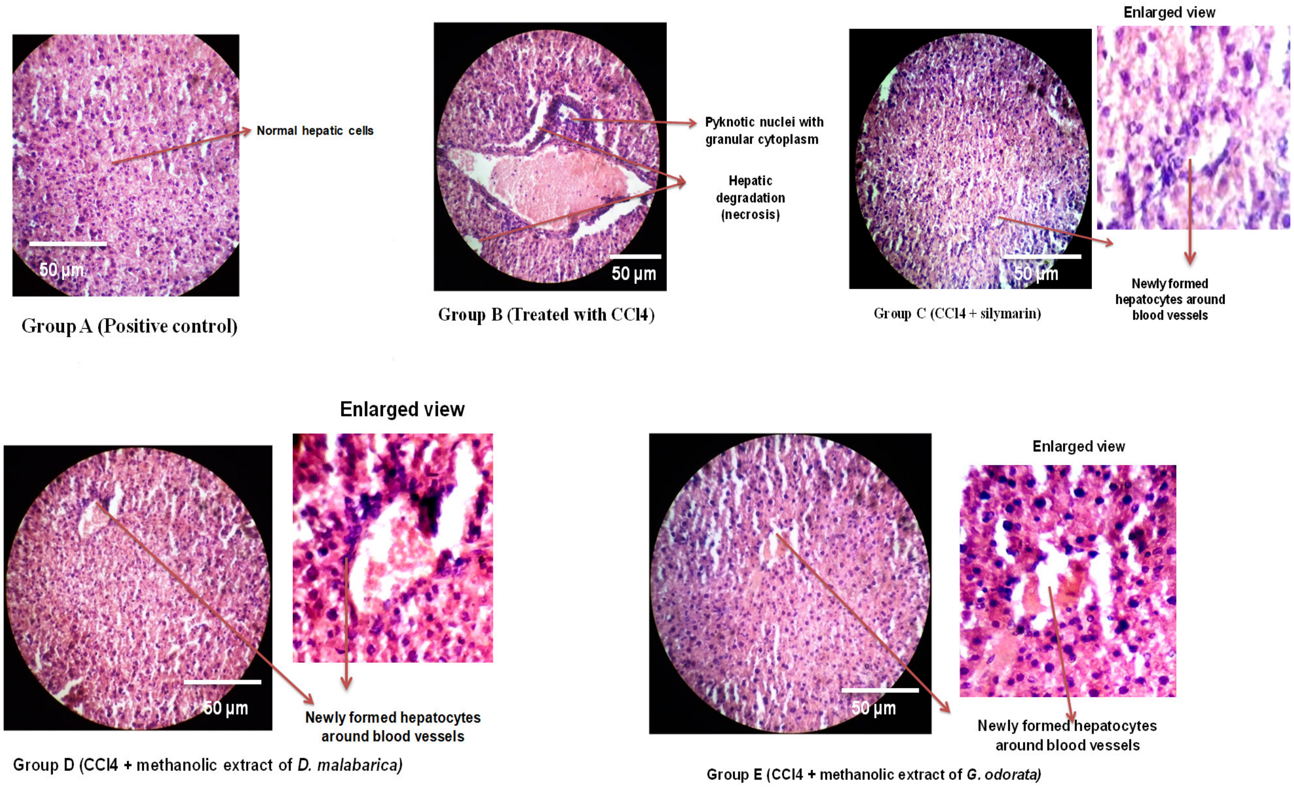

Figure S2. Histopathological studies (20 X resolution) of liver from Wister rat (A) transverse section of the liver (Goup A) showed healthy tissues, (B) hepatocytes displayed the appearance of polynuclei with granular cytoplasm in the negative control (Group B) treated with CCl_4, (C) regeneration of hepatocytes around the blood vessels was observed in the silymarin treated liver, (D) newly formed hepatocytes around the blood vessels and comparatively low granular structures in quercetin treated in Groups D and Group E rats.

Supplementary (ST)

Table ST1. Phytochemicals in the ethanolic extracts of fruit from *D. malabarica* and *G. odorata*

Phytochemicals	*D. malabarica* Presence(+)/Absence(-)	*G. odorata* Presence(+)/Absence(-)
Flavonoids	+	+
Tannin	+	+
Saponin	+	+
Ascorbic acid	+	+
Alkaloids	+	+

Table ST2. The quantitative analysis of different biochemicals present in ethanolic extracts of fruit from *D. malabarica* and *G. odorata*

**Sl. No.**	**Phytochemical**	***D. malabarica***	***G. odorata***
1	Carbohydrate content (%)	8.56 ± 0.20	9.25 ± 0.38
2	Protein content (%)	4.77 ± 0.17	3.52 ± 0.24
3	Phenolic content (µgGAE/mg)	223.5 ± 0.26	206.14 ± 0.52
4	Ascorbic acid (mg/100g)	55.57 ± 0.75	42.66 ± 0.83

All samples are measures in triplicates, values are in mean ± SD
