# Supplementary material for: Nonlinear molecular dynamics of quercetin in Gynocardia odorata and Diospyros malabarica fruits: Its mechanistic role in hepatoprotection
Source: PLoS One. 2022 Mar 21;17(3):e0263917. doi: 10.1371/journal.pone.0263917 (PMC8936497; doi:10.1371/journal.pone.0263917)
Supplement: S2 File — (PDF) [file pone.0263917.s002.pdf]

Electronic supplementary file 2

Nonlinear Molecular Dynamics of Quercetin in *Gynocardia odorata* and *Diospyros malabarica* fruits: Its mechanistic role in hepatoprotection

Arabinda Ghosh[1†], Pranjal Sarmah[2†], Harun Patel[3], Nobendu Mukerjee[4], Rajbardhan Mishra[5], Saad Alkahtani[6]**, Rajender S.Varma[7], Debabrat Baishya[2]*

Group A

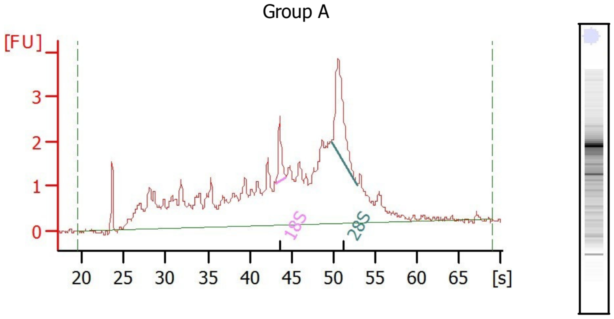

Overall Results for sample 1 : **G A**

RNA Area:	71.4
RNA Concentration:	248 ng/µl
rRNA Ratio [28s / 18s]:	3.3
RNA Integrity Number (RIN):	4.2 (B.02.08)
Result Flagging Color:	
Result Flagging Label:	RIN: 4.20

Fragment table for sample 1 : **G A**

Name	Start Time [s]	End Time [s]	Area	% of total Area
18S	43.10	44.35	1.5	2.1
28S	49.75	52.75	4.8	6.7

Group B

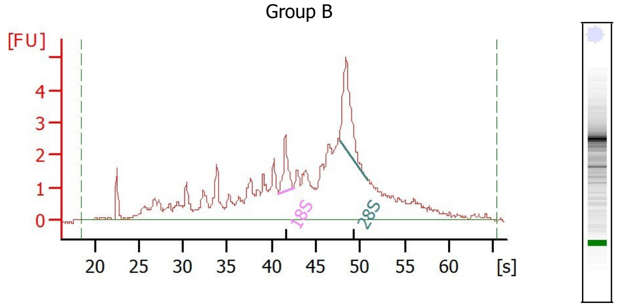

Overall Results for sample 2 : **G B**

RNA Area:	83.5
RNA Concentration:	291 ng/µl
rRNA Ratio [28s / 18s]:	2.5
RNA Integrity Number (RIN):	4.5 (B.02.08)
Result Flagging Color:	
Result Flagging Label:	RIN: 4.50

Fragment table for sample 2 : **G B**

Name	Start Time [s]	End Time [s]	Area	% of total Area
18S	40.78	42.44	2.4	2.9
28S	47.66	50.84	6.2	7.4

Group C

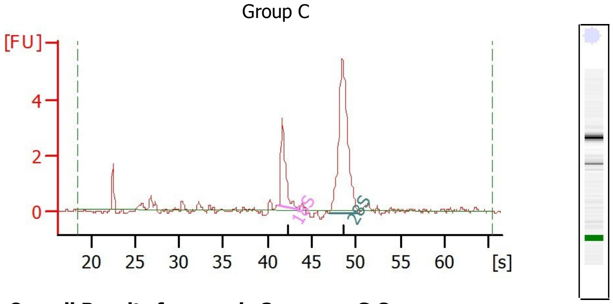

Overall Results for sample 3 : **G C**

RNA Area:	22.4
RNA Concentration:	78 ng/µl
rRNA Ratio [28s / 18s]:	3.0
RNA Integrity Number (RIN):	8 (B.02.08)
Result Flagging Color:	
Result Flagging Label:	RIN:8

Fragment table for sample 3 : **G III**

Name	Start Time [s]	End Time [s]	Area	% of total Area
18S	41.25	43.53	4.6	20.3
28S	47.09	50.27	13.5	60.4

Group D

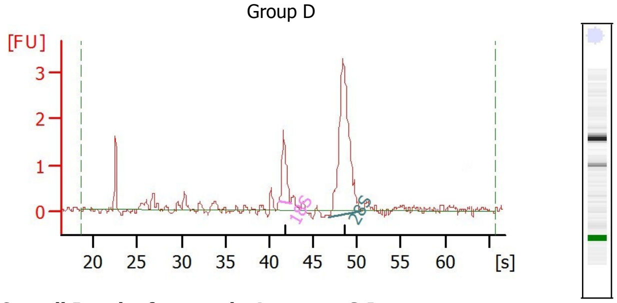

Overall Results for sample 4 : **G D**

RNA Area:	16.6
RNA Concentration:	58 ng/µl
rRNA Ratio [28s / 18s]:	4.4
RNA Integrity Number (RIN):	7.4 (B.02.08)
Result Flagging Color:	
Result Flagging Label:	RIN: 7.40

Fragment table for sample 4 : **G IV**

Name	Start Time [s]	End Time [s]	Area	% of total Area
18S	41.09	42.43	2.0	12.3
28S	46.76	50.29	8.9	53.6

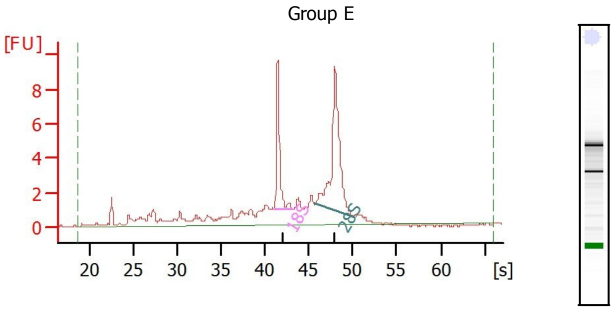

Overall Results for sample 5 : G E

RNA Area:	73.5
RNA Concentration:	256 ng/µl
rRNA Ratio [28s / 18s]:	2.2
RNA Integrity Number (RIN):	7.5 (B.02.08)
Result Flagging Color:	
Result Flagging Label:	RIN: 7.50

Fragment table for sample 5 : G V

Name	Start Time [s]	End Time [s]	Area	% of total Area
18S	41.07	43.23	8.2	11.2
28S	45.67	50.07	17.8	24.2

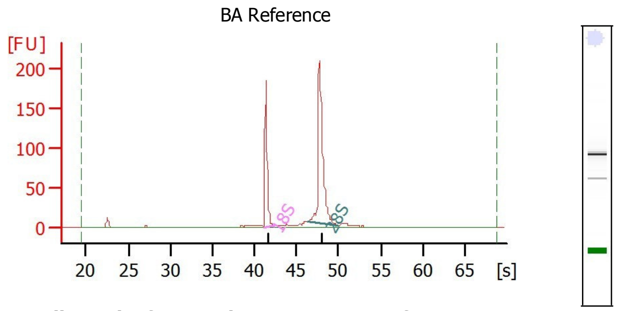

Overall Results for sample 6 : BA Reference

RNA Area:	533.0
RNA Concentration:	489 ng/µl
rRNA Ratio [28s / 18s]:	1.9
RNA Integrity Number (RIN):	10 (B.02.08)
Result Flagging Color:	
Result Flagging Label:	RIN:10

Fragment table for sample 6 : BA Reference

Name	Start Time [s]	End Time [s]	Area	% of total Area
18S	41.06	42.31	140.5	26.4
28S	46.35	49.89	269.8	50.6

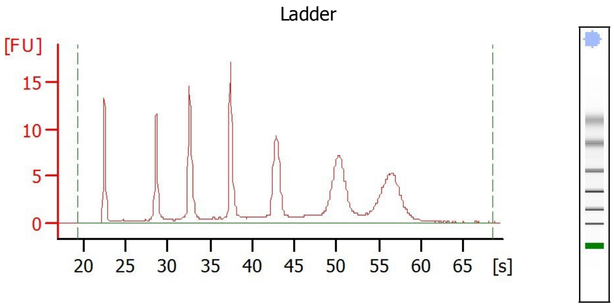

Overall Results for sample 7 : Ladder

RNA Area:	163.5
RNA Concentration:	150 ng/µl
Result Flagging Color:	
Result Flagging Label:	All Other Samples

Fragment table for sample 7 : Ladder

Name	Start Time [s]	End Time [s]	Area	% of total Area
18S	41.06	42.31	140.5	26.4
28S	46.35	49.89	269.8	50.6

Fig. S3. Qualitative and quantitative assessment of purified RNA from each individual groups GA-GE.

Supplementary Table (ST)

Table ST3. Quality assessment of RNA purification from the liver tissue sample having purity, yield and concentration.

SL.No	Sample ID	260/280	260/230	ng/ul	Yield (ng)	QC Purity	QC Conc./Yield
1	GroupA (GA)	2.14	2.07	610.8	12216	Optimal	Optimal
2	GroupB (GB)	2.08	1.23	564.4	11288	Optimal	Optimal
3	GroupC (GC)	2.13	1.4	208.4	4168	Optimal	Optimal
4	GroupD (GD)	2.13	0.85	334.6	6692	Optimal	Optimal
5	Group E (GE)	2.13	2.16	882.3	17646	Optimal	Optimal
